# Distribution of insulin in trigeminal nerve and brain after intranasal administration

**DOI:** 10.1038/s41598-019-39191-5

**Published:** 2019-02-22

**Authors:** Jeffrey J. Lochhead, Kathryn L. Kellohen, Patrick T. Ronaldson, Thomas P. Davis

**Affiliations:** Department of Pharmacology, University of Arizona College of Medicine, Tucson, AZ 85724-5050 USA

## Abstract

In the brain, insulin acts as a growth factor, regulates energy homeostasis, and is involved in learning and memory acquisition. Many central nervous system (CNS) diseases are characterized by deficits in insulin signaling. Pre-clinical studies have shown that intranasal insulin is neuroprotective in models of Alzheimer’s disease, Parkinson’s disease, and traumatic brain injury. Clinical trials have also shown that intranasal insulin elicits beneficial cognitive effects in patients with Alzheimer’s disease. It is known that insulin can be detected in the CNS within minutes following intranasal administration. Despite these advances, the anatomical pathways that insulin utilizes to reach the CNS and the cellular CNS targets after intranasal administration are not fully understood. Here, we intranasally administered fluorescently labeled insulin and imaged its localization within the brain and trigeminal nerves. Our data indicates that intranasal insulin can reach cellular CNS targets along extracellular components of the trigeminal nerve. Upon CNS entry, we found insulin significantly increased levels of an activated form of the insulin receptor. These findings suggest that the intranasal route of administration is able to effectively deliver insulin to CNS targets in a biologically active form.

## Introduction

Reduction in brain insulin signaling and/or insulin resistance is a prominent feature of type 2 diabetes mellitus, obesity, Alzheimer’s disease, and Parkinson’s disease^1–4^. Activation of insulin receptors in the brain has been shown to positively affect learning and memory, increase energy metabolism, and provide neuroprotection through the PI3K/Akt and/or MAPK signaling pathways in numerous CNS disease models^5,6^. These observations have led to the hypothesis that restoring brain insulin signaling could be an effective treatment for deficits associated with neurological diseases such as dementia, stroke, traumatic brain injury, and Parkinson’s disease^7–12^. Intranasal (IN) insulin has been shown to improve memory in healthy humans and in a clinical trial to treat patients with Alzheimer’s disease, IN insulin improved cognitive function and preserved metabolic integrity^13,14^.

Advantages of IN drug delivery, over other routes of administration, include non-invasiveness, ease of self-administration, rapid absorption and onset of action, and avoidance of hepatic first-pass elimination^15^. Due to its low bioavailability (~3–8% compared to an intravenous bolus injection), plasma levels of intranasally administered insulin are unlikely to cause systemic side effects such as hypoglycemia^16–18^. The AUC_brain:plasma_ ratio is approximately 2000-fold higher following IN delivery as compared with subcutaneous administration of insulin, suggesting that the IN route is able to preferentially target insulin to the brain^19^.

It has previously been shown that intranasally administered peptides or proteins can bypass the blood-brain barrier (BBB) to reach the brain along direct pathways connecting the nasal lamina propria with the CNS^15^. Thorne *et al*. showed that intranasally administered [^125^I]-insulin-like growth factor I (IGF-I) is delivered to the brain along components associated with the olfactory and trigeminal nerves^20^. Perivascular distribution in brain tissue can be observed within minutes following IN administration of fluorescently labeled dextrans^21^. This observation is critical to understanding IN drug delivery because it indicates that bulk flow within cerebral perivascular spaces can facilitate rapid, widespread brain delivery of intranasally administered therapeutics.

Insulin has previously been detected in the cerebrospinal fluid (CSF) of humans and in brain regions such as the olfactory bulbs, hippocampus, cortex, cerebellum, brain stem, and hypothalamus of rodents within minutes following IN administration^7,17,22,23^. These studies provided valuable quantitative data showing delivery of IN insulin to the CNS, but the methodologies used were not conducive to showing qualitative distribution of insulin within the tissues examined. Renner *et al*. previously showed distribution of fluorescently labeled insulin within the olfactory nerves and olfactory bulb after IN administration, but insulin localization within the trigeminal nerve and other brain areas was not shown^24^. In the present study, we intranasally administered fluorescein isothiocyanate (FITC)-insulin and examined its distribution within the brain and trigeminal nerves. Our novel data suggest that insulin can reach the brain along perineural spaces of the trigeminal nerve, distribute along cerebral perivascular spaces, and activate brain insulin receptors after IN administration.

## Results

### FITC-insulin distribution in the trigeminal nerve after IN administration

**Figure 1:**
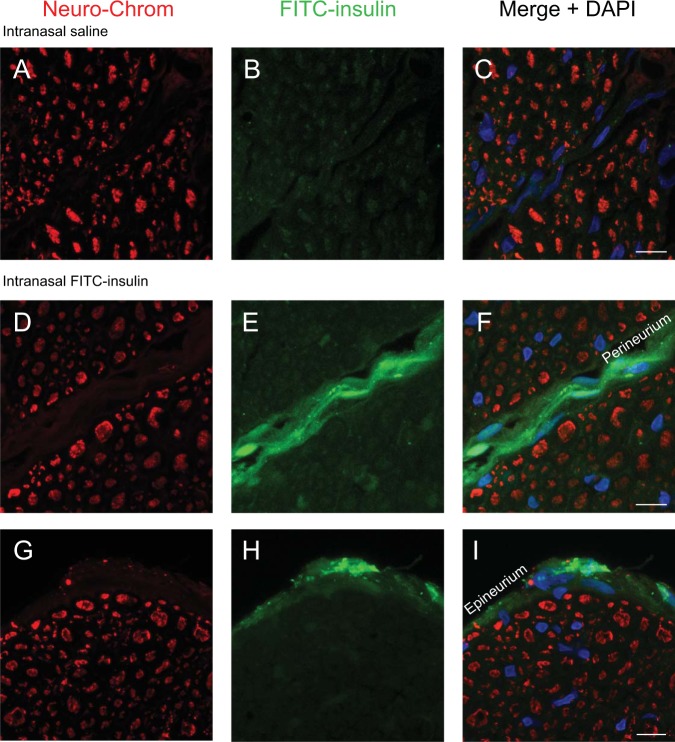
FITC-insulin was intranasally administered and imaged in the trigeminal nerve 30 min later (**B**,**E**,**H**). Axons in the trigeminal nerve were labeled with the pan-neuronal marker Neuro-Chrom (**A**,**D**,**G**). The merged images (**C**,**F**,**I**) show FITC-insulin in the perineural spaces of the trigeminal nerve. These data suggest FITC-insulin reaches the CNS along perineural spaces of the trigeminal nerve. Scale bar = 10 μm.

The trigeminal nerves innervate the nasal passages from the brain and have previously been implicated as a pathway to intranasally deliver drugs to the CNS^20^. Trigeminal nerves were dissected from the base of the skull. The region excised was between where the nerve emerges directly from the brain and where the V_1_V_2_ branches exit the cranial compartment through the anterior lacerated foramen. Cross sections of the trigeminal nerves were examined approximately 30 min after IN administration of saline (Fig. 1B) or FITC-insulin (Fig. 1E,H). Neuronal cell bodies and axons were labeled with the Neuro-Chrom antibody (Fig. 1A,D,G). Merged images (Fig. 1C,F,I) show a faint signal of FITC-insulin localized in the perineural spaces of the endoneurium (space between individual axons) and a more prominent signal associated with the perineurium (space between axon bundles) and epineurium (space in outermost layer of nerve) of the trigeminal nerve. These data suggest insulin is able to reach the brain via the nasal passages along extracellular perineural spaces of the trigeminal nerve.

### FITC-insulin can be found associated with cerebral blood vessels after IN administration

**Figure 2:**
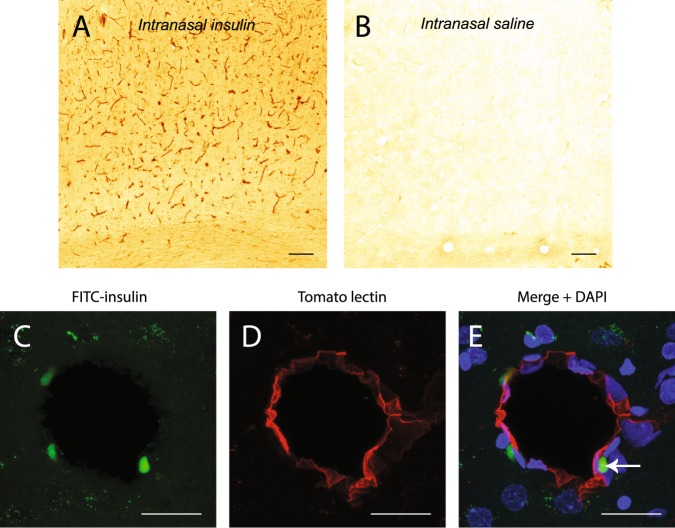
FITC-insulin can be detected with an anti-insulin antibody and shows widespread association with blood vessels in the cortex of rats after IN administration (**A**). Little staining is observed in rats administered IN saline (**B**). FITC-insulin is observed in the perivascular space (**C**) of cortical blood vessels (**D**) 30 min after intranasal administration. Merged image + DAPI is shown in (**E**). Scale bar = 100 μm for (**A**,**B**); 20 μmm for (**C**–**E)**.

We previously showed that fluorescently labeled dextrans rapidly distribute along cerebral perivascular spaces following IN administration^21^. To determine if insulin utilizes these same extracellular pathways, FITC-insulin distribution was examined in the cortex of rats with an anti-insulin antibody approximately 30 min after IN administration (Fig. 2A). Widespread distribution of insulin was observed associated with blood vessels. IN saline administration showed little staining with an anti-insulin antibody (Fig. 2B). Consistent with the idea that cerebral perivascular spaces are involved in brain distribution of intranasally administered macromolecules, we observed a punctate signal of FITC-insulin (Fig. 2C,E) proximal to cerebral blood vessels labeled with tomato lectin (Fig. 2D,E). This signal likely represents FITC-insulin that has been retained within perivascular spaces following perfusion-fixation or FITC-insulin that has been endocytosed by perivascular cells in the brain.

### FITC-insulin reaches cells in widespread brain areas after IN administration

**Figure 3:**
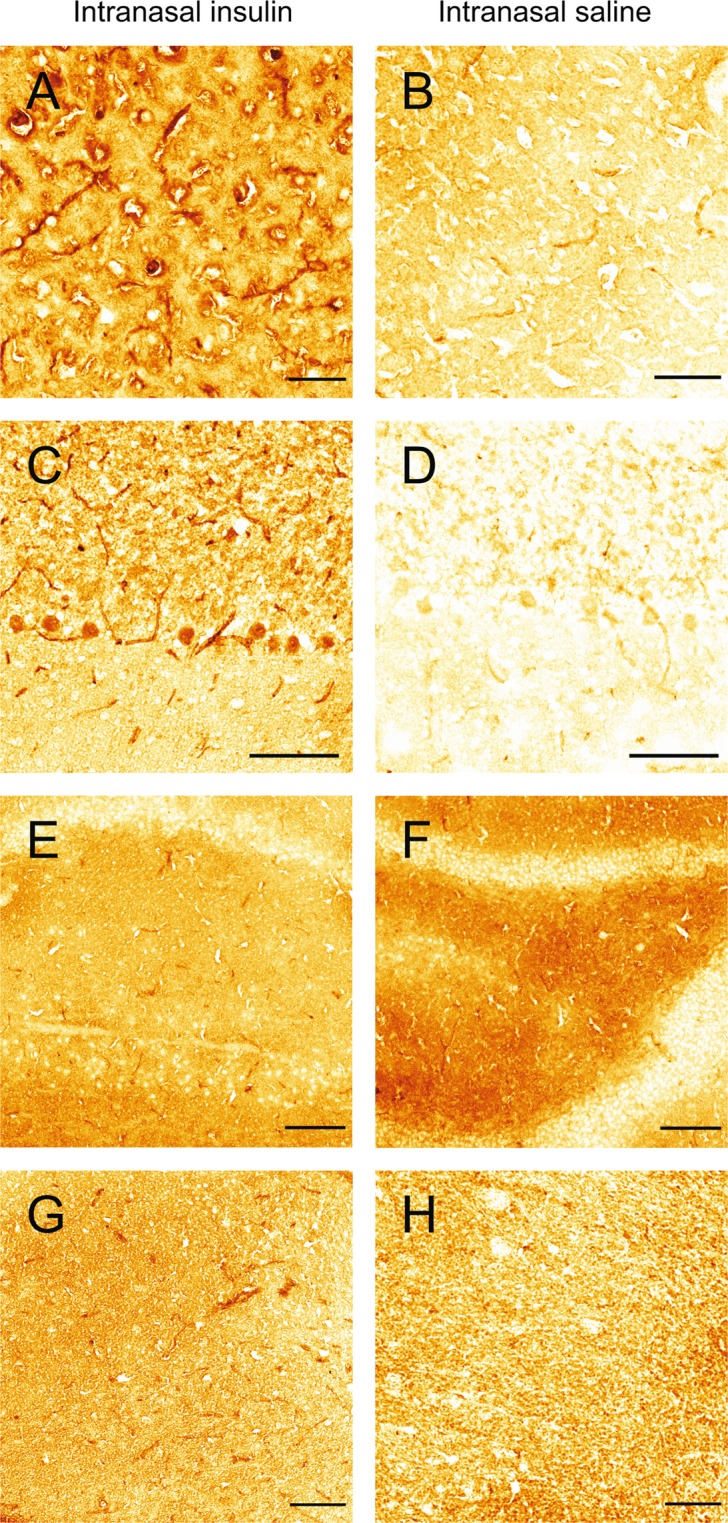
Intranasally administered FITC-insulin can be detected with an anti-insulin antibody in brain regions with high expression of insulin receptors such as the cortex (**A**), cerebellum (**C**), hippocampus (**E**), and hypothalamus (**G**). Detection of insulin in rats administered intranasal saline is reduced in the cortex (**B**) cerebellum (**D**), hippocampus (**F**), and hypothalamus (**H**). Scale bar (**A**,**B**) = 50 µm; (**C**–**H**) = 100 µm.

Next, we examined FITC-insulin distribution in brain tissue 30 min after IN delivery. In order to avoid previously identified issues associated with green cellular autofluorescence in brain^25^, we detected insulin with an anti-insulin antibody and standard immunohistochemical methods. After IN FITC-insulin administration, we observed insulin in brain areas with high expression levels of the insulin receptor (IR) such as the cortex (Fig. 3A), cerebellum (Fig. 3C), hippocampus (Fig. 3E), and hypothalamus (Fig. 3G). Rats administered IN saline showed reduced staining in the cortex (Fig. 3B), cerebellum (Fig. 3D), hippocampus (Fig. 3F), and hypothalamus (Fig. 3H). Staining observed in saline treated rats likely represents endogenous brain insulin. These data suggest IN insulin is delivered to brain areas that are critical for CNS insulin signaling within 30 min of administration.

### Insulin activates insulin receptors (IR) in brain after IN administration

**Figure 4:**
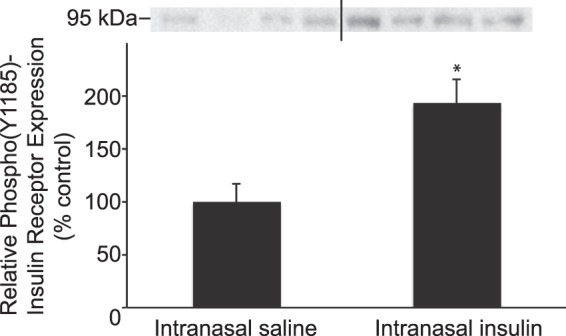
IN administration of insulin increases levels of phospho(Y1185)-insulin receptor in brain. One hour after IN administration of insulin or saline, Western blot analysis with an antibody specific for phospho(Y1185) insulin receptor was performed on whole brain homogenates. Rats administered IN insulin had significantly higher levels of phospho(Y1185)-insulin receptor in brain compared to rats administered IN saline. Relative expression levels are presented as percentage vs. control. *P < 0.02 (*n* = 4). The full-length blot is presented in Supplementary Fig. 2.

Additionally, we examined if IN insulin is able to activate IRs in brain by comparing levels of the phosphorylated (i.e. activated) β-subunit of the IR in the brains of rats administered IN saline or insulin. Binding of insulin to the IR, a transmembrane tyrosine kinase that consists of two α-subunits and two β-subunits, causes the receptor to undergo a conformational change. This conformational change activates the tyrosine kinase activity of the β-subunits, resulting in receptor auto-phosphorylation and subsequent phosphorylation of intracellular insulin receptor substrate proteins on tyrosine residues^26^. It has previously been demonstrated that auto-phosphorylation of tyrosine residue 1185 is an early event associated with insulin binding to the IR and activating intracellular signaling pathways^27,28^. We detected an activated form of the insulin receptor in brain with a primary antibody specific to the phospho(Y1185)-insulin receptor (Fig. 4). One hour after IN administration, we observed significantly higher levels (1.93-fold) of phospho(1185)-insulin receptor in the brains of rats administered IN insulin vs. IN saline using Western blot analysis (*P* < 0.02; *n* = 4). This data suggests insulin reaches the brain in an intact, biologically active form capable of binding to and activating insulin receptors after IN administration.

### SDS-PAGE Electrophoresis of FITC-insulin

**Figure 5:**
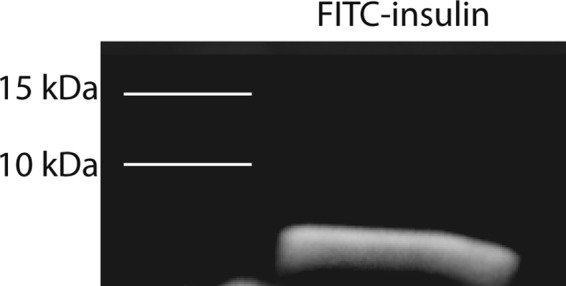
FITC-insulin (~6 kDa) was subjected to SDS-PAGE on a 4–15% Criterion TGX gel (BioRad) and imaged on a ChemiDoc Touch Imaging System (BioRad) to confirm the FITC-insulin used in the study is primarily monomeric. The full-length gel is presented in Supplementary Fig. 1.

In solution, insulin can aggregate into dimers and hexamers that may potentially affect the efficiency of drug delivery to the brain^29,30^. We subjected the FITC-insulin (~6 kDa) used in this study to SDS-PAGE electrophoresis to allow us to visualize whether the insulin was primarily monomeric, dimeric, or hexameric. We detected no bands on the gel >10 kDa, suggesting the FITC-insulin used in the study was primarily monomeric (Fig. 5).

## Discussion

Insulin is increasingly being used as a therapeutic in pre-clinical and clinical studies of neurological disorders due to its ability to provide neuroprotection, increase synaptogenesis, improve learning and memory, and positively regulate energy homeostasis^3,4,6,8,13^. Beneficial effects of IN insulin on learning and memory have been observed in healthy humans and Alzheimer’s disease patients and clinical trials will determine the effectiveness of IN insulin to treat Parkinson’s disease, multiple sclerosis, ischemic stroke, depression, and Gulf War multisymptom illness (clinicaltrials.gov). Intranasal application is the preferred route of administration to deliver insulin to the brain due to its potential to bypass the blood-brain barrier and its high AUC_brain:plasma_ compared to intravenous or subcutaneous administration, which reduces off-target effects such as insulin-induced hypoglycemia^7,17–19^.

Previously it has been shown that [^125^I]-IGF-I is able to reach the brain along components of the olfactory and trigeminal nerves following IN administration^20^. After entering the brain, we have shown that intranasally administered fluorescent dextrans exhibit widespread distribution along cerebral perivascular spaces^21^. Several studies have quantified brain uptake of IN insulin^7,17,19^. These studies are limited because they did not address whether: (i) IN insulin reaches the brain along the same pathways as IGF-I and (ii) IN insulin distributes along cerebral perivascular spaces. In the present study, we have attempted to address these knowledge gaps by examining distribution of FITC-insulin in brain tissue and in the trigeminal nerve after IN administration. We chose to administer a dose corresponding to 6 Units of insulin because this dose has previously been shown to deliver insulin to the brain within 45 min and provide beneficial treatment in a pre-clinical model of traumatic brain injury^7^. This dose represents a human equivalent dose approximately 6-fold higher than the maximum dose administered to patients in a phase II trial to treat Alzheimer’s disease, although species differences in relative surface area of the nasal cavity and brain size should be considered when converting intranasal doses between rats and humans^14,31^.

Renner *et al*. have shown that Alexa Fluor 647-insulin is able to rapidly reach the olfactory bulbs along the olfactory nerves that enter the brain through the cribriform plate from the nasal lamina propria^24^. Our data extends upon these findings and shows localization of FITC-insulin in the perineural spaces of the endoneurium, perineurium, and epineurium of the trigeminal nerve, with the endoneurium showing only faint levels of fluorescence. In both humans and rodents, branches of the trigeminal nerve (and its associated arteries) form anatomical connections between the brainstem and nasal lamina properia^15^. These connections provide potential brain entry points for drugs from the nasal lamina propria.

Renner *et al*. reported no detection of Alexa Fluor 647-insulin in the trigeminal nerve after IN administration^24^. Differences between their study and our study may be due to the insulin used. Renner *et al*. administered an insulin solution consisting of predominantly dimers. The FITC-insulin used in our study was monomeric (Fig. 5). We have previously shown that the nasal epithelium is more permeable to 3 kDa dextrans than 10 kDa dextrans^15^. The ~14 kDa insulin dimers used by Renner *et al*. may have been less permeable across the nasal epithelium than the ~6 kDa monomers used in our study. A lower permeability of insulin dimers across the nasal epithelium would likely restrict access to components of the trigeminal nerve in the nasal lamina propria and result in a lower or undetectable fluorescent signal. Insulin has a propensity to aggregate in solution and our data suggests insulin monomers may reach the CNS at higher levels than dimers^30^. Our findings and the data from Renner *et al*., suggests IN insulin is able to reach the brain along extracellular spaces of the olfactory and trigeminal nerves in the rostral to caudal direction. Salameh *et al*. have shown that protein kinase C (PKC) inhibition results in higher brain delivery of insulin after IN administration^17^. It is unknown whether PKC inhibition modifies permeability of the nasal epithelium, affects insulin entry into perivascular and/or perineural spaces, or works by an unrelated mechanism.

Insulin that has been absorbed into the systemic circulation after administration may also reach the brain by crossing the BBB or blood-CSF barrier (BCSFB). Insulin crosses the BBB by a saturable transcellular transport mechanism^32^. Insulin receptors are present at the BBB and the BCSFB and have been proposed to mediate transport of insulin from the blood to the CNS, although some studies have shown expression of the insulin receptor at the BBB is not necessary for insulin transport^33–37^. Whether IN insulin measured in the CSF crosses the BCSFB from the systemic circulation or originates from the olfactory or trigeminal pathways in the nasal lamina propria warrants further investigation. Higher AUC_brain:plasma_ following IN versus subcutaneous or intravenous administration suggests most of the insulin reaching the brain after IN administration is through direct pathways connecting the nasal lamina propria to the CNS (i.e. olfactory and trigeminal pathways), rather than through the BBB or BCSFB from the plasma. A higher AUC_brain:plasma_ after IN administration suggests the IN route would have a more favorable safety profile in contrast with other routes of administration for insulin.

Fluorescent tracers injected into the cisterna magna distribute into the cerebral perivascular spaces^38,39^ and the FITC-insulin we observed in the perivascular space likely originated from either the CSF, from the perivascular spaces of arteries associated with the olfactory and trigeminal nerves, or insulin which has been transported across the BBB after absorption into the bloodstream. Any of these routes may allow intranasally administered insulin to distribute throughout the brain.

After IN administration, we observed FITC-insulin delivery to brain cells in areas of the brain exhibiting high expression of insulin receptors such as the hippocampus, hypothalamus, cortex, and cerebellum^40^. In addition, we observed significantly increased levels of an activated form of the insulin receptor (i.e. phosphorylated on tyrosine residue 1185) in brain homogenates, suggesting IN administration delivers insulin to the brain in a biologically active form. In summary, our data suggests intranasally administered insulin is able to reach the brain along perineural spaces of the trigeminal nerve and is able to reach widespread brain areas important for insulin signaling. Future studies will need to address whether disease states may impact the efficiency of IN drug delivery to the CNS. It has been shown that perivascular transport is altered or impaired in aging as well as in animal models of microinfarcts and traumatic brain injury^41–44^. Pathophysiological changes in cerebral perivascular transport may impede delivery of insulin and other therapeutics to the brain via the IN route. An improved understanding of mechanisms associated with IN delivery of therapeutics to the CNS under normal and pathophysiological conditions may lead to more effective methods to treat neurological disorders.

## Methods

### Reagents

Human insulin (Humulin R) was purchased from Eli Lily. FITC-insulin was purchased from Sigma-Aldrich. Vectastain Universal Elite ABC kit, Carbo-Free blocking solution, and DyLight 649-tomato lectin were purchased from Vector Laboratories. Bovine serum albumin (BSA) was purchased from Rockland Immunochemicals. Phospho-insulin receptor beta (Y1185) antibody was purchased from Bioss (#bs-5453R). Rabbit anti-insulin antibody and horseradish peroxidase conjugated anti-rabbit IgG antibody was purchased from Cell Signaling Technology. Neuro-Chrom pan-neuronal antibody was purchased from Millipore (#ABN2300). Phosphatase inhibitor cocktail was purchased from Research Products International. Complete mini protease inhibitor cocktail was purchased from Roche. SuperBlock blocking buffer, 4,6-diamidino-2-phenylindole (DAPI), IP lysis buffer, ProLong Diamond, and Alexa Fluor 568 goat anti-mouse antibody were purchased from Thermo Fisher. Any KD TGX gels, XT sample buffer, reducing agent, and Clarity Western ECL reagent were purchased from Bio-Rad. All other reagents were purchased from Sigma-Aldrich unless noted.

### Animals and treatment

All experimental protocols were approved by the Institutional Animal Care and Use Committee at the University of Arizona in accordance with National Institutes of Health guidelines. Female Sprague Dawley rats (200–250 g; Envigo) were housed under a 12 h light/dark cycle and fed *ad-libitum*. Food was withheld from rats 12–18 h before administration of insulin. Prior to IN administration, rats were anesthetized with an intraperitoneal injection of ketamine/xylazine (100 mg/kg K: 10 mg/kg X). Rats were placed in the supine position on a heating pad maintained at 37 °C and one 20 µl drop of insulin or FITC-insulin (total dose ~6 Units/40 µl saline) was pipetted into each naris 5 min apart. Controls were administered 40 µl saline. Thirty or 60 minutes after the first drop was intranasally administered, rats were transcardially perfused with 0.01 M phosphate buffered saline (PBS) followed by ~100 ml 4% paraformaldehyde in 0.1 M phosphate buffer (4% PFA) or ~250 ml HistoChoice Tissue Fixative. The brain and trigeminal ganglia were removed and post-fixed overnight in 4% PFA or HistoChoice at 4 °C. Tissue was then placed in 20% sucrose in PBS overnight followed by overnight immersion in 30% sucrose in PBS at 4 °C. The tissue was then snap-frozen in isopentane on dry ice and 10–20 µm-thick cryosections were mounted onto glass slides for staining. To obtain brain homogenates, rats were transcardially perfused with PBS 1 h after intranasal administration of insulin. Brains were removed and homogenized in ice-cold IP lysis buffer (8 ml) with added phosphatase and protease inhibitors. Brain homogenates were centrifuged at 13,000 × *g* for 20 min and the supernatant was stored at −20 °C for Western blot analysis.

### Immunostaining

Brain sections fixed in PFA were incubated for 30 min in Carbo-Free blocking solution followed by 30 min in DyLight 649-Tomato lectin (1:1000, Vector Labs), washed and then coverslipped in ProLong Diamond for confocal microscopy. Trigeminal nerves fixed in PFA were blocked in PBS with 0.3% Tx-100 + 5% BSA and incubated in Neuro-Chrom primary antibody (1:1000) overnight at 4 °C. Sections were then washed and incubated in Alexa Fluor 568 goat anti-mouse secondary antibody (1:500) for 1 h at room temperature. Sections were washed and nuclei were stained with DAPI. Sections were then coverslipped in ProLong Diamond for confocal microscopy. Brain sections fixed in HistoChoice were blocked in PBS with 0.05% Tween-20 + 1% BSA for 1 h and then incubated in rabbit anti-insulin antibody (1:50) overnight at 4 °C. Standard ABC methods following the manufacturer’s instructions were then utilized to detect insulin using 3,3-diamonobenzidine (DAB) as the chromogenic substrate using light microscopy.

### Microscopy

Confocal or light microscopy was performed on a Leica SP8 confocal microscope. Images from control and treated animals were acquired using the same settings. Adjustments for brightness and contrast levels were performed with FIJI (NIH) or Photo Shop (Adobe Systems) in an identical manner for both control and treated images.

### SDS-PAGE electrophoresis

Freshly prepared FITC-insulin dissolved in saline was added to XT sample buffer and XT reducing agent and electrophoresed on a 4–15% TGX gel after heating to 70 °C for 10 min. The gel was then imaged in a ChemiDoc Touch Imaging system under the fluorescein setting.

### Western blot analysis

Whole brain homogenates were added to XT sample buffer and XT reducing agent and heated at 70 °C for 10 min. Equal amounts of protein (determined by BCA protein assay) from brain homogenates of rats treated with IN saline or IN insulin were loaded onto Any KD TGX gels and subjected to SDS-PAGE. Protein was then transferred onto a polyvinylidene difluoride membrane and blocked for 1 h at room temperature in SuperBlock blocking buffer with 0.05% Tween-20. Membranes were incubated overnight at 4 °C in blocking buffer with rabbit anti-phospho(Y1185)-insulin receptor (1:1000). Membranes were washed and incubated in HRP-conjugated anti-rabbit IgG secondary antibody (1:2000) for 1 h at room temperature. Bands were visualized using ECL on a Bio-Rad ChemiDoc imaging system. Because insulin has previously been shown to alter common housekeeping genes used as loading controls such as actin, tubulin, and GAPDH, the optical density of the bands was quantified using FIJI and normalized to a total protein stain according to previously published methods^45–51^.

### Statistical analysis

A Student’s T-test was used to detect differences between treatment groups using Graph Pad software. Differences were considered statistically significant if P < 0.05.

## Supplementary information

Supplementary Dataset 1

## Data Availability

The datasets generated during and/or analysed during the current study are available from the corresponding author on reasonable request.
