# Supplementary material for: Distribution of insulin in trigeminal nerve and brain after intranasal administration
Source: Sci Rep. 2019 Feb 22;9:2621. doi: 10.1038/s41598-019-39191-5 (PMC6385374; doi:10.1038/s41598-019-39191-5)
Supplement: Supplementary file 1 — Supplementary Dataset 1 [file 41598_2019_39191_MOESM1_ESM.pdf]

Distribution of insulin in trigeminal nerve and brain after intranasal administration

Jeffrey J. Lochhead, Kathryn L. Kellohen, Patrick T. Ronaldson, Thomas P. Davis

A FITC-insulin

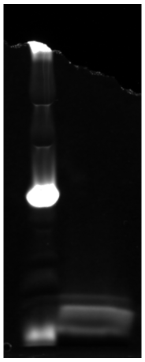

B Phospho(Y1185)-insulin receptor

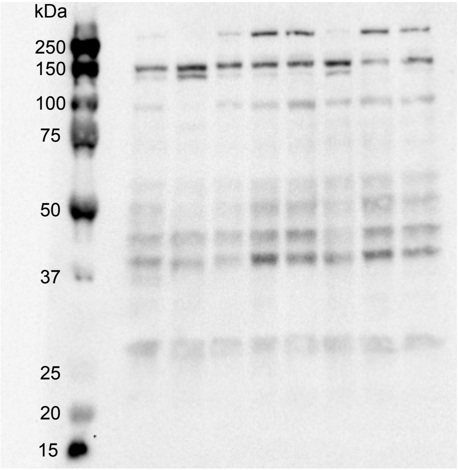

Supplementary Figure 1 - Full length images of the gel which FITC-insulin was run on (A) and the Western blot analyzed for phospho(Y1185) insulin receptor expression (B).
